# Generating geochemical and mineralogy distributions of soil in the conterminous United States using Bayesian hierarchical spatial models

**DOI:** 10.1016/j.mex.2026.103836

**Published:** 2026-02-19

**Authors:** Kristin J. Bondo, Tiffany M. Wolf, W. David Walter

**Affiliations:** a Department of Veterinary Population Medicine, University of Minnesota, St. Paul, MN 55108, USA; b Minnesota Center for Prion Research and Outreach (MNPRO), University of Minnesota, St. Paul, MN 55108, USA; c U.S. Geological Survey, Pennsylvania Cooperative Fish and Wildlife Research Unit, The Pennsylvania State University, University Park, PA, 16802, USA

**Keywords:** Bayesian hierarchical model, element, geochemistry, integrated nested Laplace approximation, mineral, spatial model, stochastic partial differential equation

## Abstract

Characterizing geochemical and mineralogical soil distributions across large spatial extents is essential for understanding mineral resources, ecosystem processes, and environmental risks. Rasters of soil geochemical distributions for the conterminous United States, however, are limited. We present a Bayesian modeling workflow and tool for generating predictive geochemical and mineralogy distribution maps for the conterminous United States using integrated nested Laplace approximation (INLA) with the stochastic partial differential equation approach. By modeling soil geostatistical data with environmental covariates (soil properties, topography, climate, and land cover), we generate predictive distributions of soil geochemistry that can be mapped or extracted for further analyses. As an example, we model the spatial distribution of trace elements in soil relevant to vertebrate health (cobalt, copper, iron, manganese, selenium, and zinc) and provide a workflow that can be used to generate and visualize predictive distributions of 39 other major and trace elements and 21 minerals of the soil survey, supporting a variety of ecological, environmental, and agricultural applications.

**Bayesian Modeling:** Uses R-INLA to predict soil geochemistry across large spatial extents.

**Covariate Integration:** Incorporates environmental variables to increase predictive accuracy.

**Raster Generation:** Produces continuous geospatial layers of element and mineral distributions of the conterminous United States for a variety of applications.

## Graphical abstract

**Figure:**
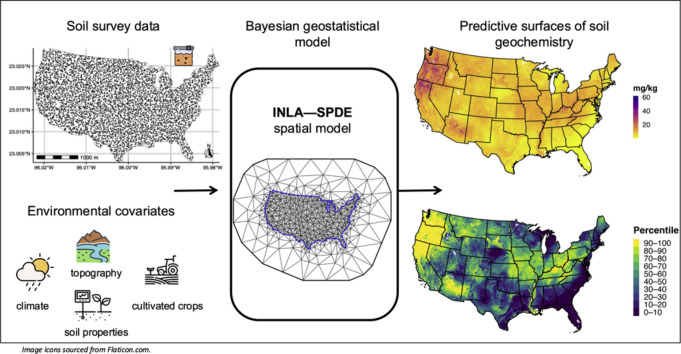

**Specifications table**

**Table:** 

**Subject area**	Agricultural and Biological Sciences
**More specific subject area**	Spatial Statistics
**Name of your method**	Mapping geochemical distributions of soil using R-INLA
**Name and reference of original method**	Mapping geochemical distributions of soil using interpolation • Smith, D.B., Cannon, W.F., Woodruff, L.G., Solano, F, and Ellefsen, K.J., 2014, Geochemical and mineralogical maps for soils of the conterminous United States: U.S. Geological Survey Open-File Report 2014–1082, 386 p, 10.3133/ofr20141082.
**Resource availability**	Information on all resources to reproduce this method are included in the present article.

## Background

Environmental geochemistry investigates factors that govern the origin, distribution, and transport of elements within the environment, focusing on their pathways into soil, food, and water, and their subsequent effects on plant and animal health [1]. Large-scale information on the distribution of naturally occurring soil elements was limited in North America until the U.S. Geological Survey (USGS) conducted a large-scale and low density (1 site per 1600 km^2^) soil sampling effort from 2007 to 2010, collecting samples from 4857 sites across the conterminous United States [[2], [3], [4], [5]]. This effort was originally part of the North American Soil Geochemical Landscapes (NASGS) project, which included as one of its initiatives to produce a comprehensive geochemical and mineralogical database for North America and visualize the data through mapped representations [6]. Publicly available data from the NASGS project is limited to the United States, as Mexico did not release its data publicly, and Canada was unable to complete the project due to shifting priorities within the Geological Survey of Canada in 2009 [7,8].

Resulting maps of the United States illustrated spatial variations in mineral and element concentrations using interpolated color surfaces and proportional symbols at a resolution of approximately 21 km corresponding to an area of 444 km^2^ [3]. While effective for visualization, these methods were inaccurate for making predictions in unsampled areas [3,9]. In addition, the downloadable files were image rasters and unsuitable for direct extraction of data for statistical modeling. Integrated nested Laplace approximation (INLA) combined with the stochastic partial differential equation (SPDE) approach using R-INLA software [[10], [11], [12], [13]] offers an efficient alternative for modeling continuous spatial processes in geostatistical data, particularly for large datasets [13,14]. Unlike traditional interpolation methods such as kriging or inverse distance weighting, which rely on predefined spatial structure assumptions, Bayesian hierarchical spatial models explicitly model the underlying spatial process while incorporating observed data and spatial dependencies [12,14]. This approach offers advantages for capturing complex spatial patterns and uncertainty, particularly in areas with sparse data [15]. Several studies found INLA-SPDE Bayesian spatial models to be equally or more robust for mapping distributions of environmental variables when compared to other methods [[16], [17], [18], [19]]. In addition, the Bayesian hierarchical modeling framework used to fit these models allows the integration of multiple environmental predictors, enhancing explanatory power beyond conventional interpolation techniques [14,20]. A further benefit is that Bayesian spatial models produce full uncertainty estimates alongside predictions, improving interpretability and confidence in results [21].

Distributions of various soil properties have been modelled and mapped using INLA-SPDE worldwide but less commonly for datasets of the United States [19,[22], [23], [24]]. Currently, comprehensive rasters of major and trace elements and minerals across the United States are not publicly available in contrast to several other soil chemical and physical properties [25]. To address this gap, we present a workflow for generating predictive geochemical and mineralogical distribution maps of soil in the conterminous United States using Bayesian hierarchical spatial models using INLA-SPDE in R [26,27]. We integrate USGS geochemical and mineralogical survey data with environmental variables (soil properties, topography, climate, and land cover) to produce ∼2.4 km resolution maps and rasters representing soil mineral and element distributions of the conterminous United States. As an example, we model distributions of trace elements relevant to vertebrate health (cobalt (Co), copper (Cu), iron (Fe), manganese (Mn), selenium (Se), and zinc (Zn)) [28] with rasters available for download [29]

Our workflow serves as a detailed semi-automated tool that can guide researchers in generating predictive distributions for all elements and minerals from the USGS soil survey dataset, including 39 other major and trace elements and 21 minerals using combinations of three different depths, supporting a variety of ecological, environmental, and agricultural applications in the conterminous United States. While general INLA methodologies to model a variety of datasets using various distributions are available [12,14,30,31], providing fully generalizable code to all datasets is challenging because practical decisions, such as mesh construction, prior selection, and model configuration, must be tailored to each dataset and research objectives. In addition, while a Bayesian stochastic soil modeling framework using INLA-SPDE is available [32], resources describing code for all steps to generate predictive rasters using INLA is more limited and for relatively smaller example datasets [33,34].

Overall, this workflow balances computational efficiency with spatial detail, producing reproducible maps and rasters of element and mineral distributions of the conterminous United States while providing a practical framework of detailed code that can be used as a resource to conduct similar analyses of other geochemical, ecological, or environmental datasets.

## Method details

### Workflow

Our workflow is organized into three R scripts that progressively prepare the dataset and fit spatial models to generate predictions of soil trace element concentrations using two distributions. This modular structure ensures reproducibility and flexibility for modeling other elements and minerals in the soil survey and adapting the workflow to other datasets or distributions. A detailed README file accompanies the code repository [26] and provides full instructions for running each step. Prior to running Script 1.0, the user must decide on a trace element to model and change the placeholder in the script to the name of the mineral, which is further described in the README file.

• **Script 1.0_Prepare_Dataset.Rmd** imports and cleans geochemical soil survey data from the USGS, extracts six trace elements (Co, Cu, Fe, Mn, Se, Zn), and generates a ∼6 km² sampling grid overlapping soil sample locations. Environmental covariates (soil properties, land use, topography) are aligned to this grid, and outputs include a cleaned dataset and standardized environmental rasters to be used in subsequent scripts.
• **Script 2.0_Predict_Mineral_Soil_Distribution_Lognormal.Rmd** fits Bayesian hierarchical spatial models to the cleaned dataset using INLA-SPDE under a lognormal likelihood, with state-level random effects. Model selection and validation guide the choice of distribution, followed by sensitivity analyses and spatial prediction on a fine-resolution grid that can be saved as a raster or plotted for visualization.
• **Script 3.0_Predict_Mineral_Soil_Distribution_Gamma.Rmd** mirrors Script 2 but applies a gamma distribution for sensitivity analyses and generating spatial predictions. While the lognormal models consistently outperformed the gamma models in our study, we include this workflow for comparison, exploratory evaluation of the modeled element distributions, and potential adaptation to other elements, minerals or datasets. This script is not further detailed in the manuscript but is provided with the code.

## Script 1.0 – Prepare dataset

### Geochemical soil survey data and sampling grid

#### Import and clean geochemical and soil survey dataset

We imported the USGS geochemical and mineralogical survey tables (top 5 cm and A horizon) into R using readxl [35]. The data cover 4857 locations (1 site per 1600 km²) across the conterminous United States (2007–2010), including samples from 0 to 5 cm, the A horizon (∼5–25 cm), and the C horizon (∼1 m) [4].

We extracted values for cobalt, copper, iron, manganese, selenium, and zinc (hereafter, trace elements) into two separate data frames using dplyr [36]. We replaced values below the lower limit of detection (LLD) with half the LLD, following [3].

#### Remove extreme outliers and missing samples

We manually identified one extreme outlier for each of cobalt, copper, selenium, and zinc based on previously generated interpolated maps [3,25], which highlighted exceptionally high concentrations likely originating from localized natural or anthropogenic sources [3]. Inclusion of these outliers was reported by [3] to generate unrealistically large areas of predicted concentrations in the interpolated maps. Thus, we programmatically removed these outlier values from the dataset in R, ensuring reproducibility and preventing them from disproportionately influencing predicted concentrations.

We excluded samples with a “LabID” of “N.S.” (not sampled). Sixteen sites in the top 5 cm and 42 sites in the A horizon had “N.S.” reported for all trace elements; for these sites, we used only the observed values in the calculations.

#### Combine soil horizons and average values

We programmatically merged the two dataframes of the top 5 cm and A horizons by site ID and averaged trace element values at each site to represent soil from approximately 0–25 cm depth, relevant to biological processes [37]. We then combined columns using *dplyr::coalesce* [36]. If predicting only the top 5 cm or A horizons are of interest, users can modify the code by skipping the merging, averaging, and coalescing steps and proceeding with the rest of the workflow using the chosen layer, updating column names and column numbers where applicable.

#### Transform coordinates and prepare sampling grid

We transformed coordinates from latitude/longitude (EPSG:4326) to a projected coordinate system (EPSG:5070) using sf [38,39]. We prepared a sampling grid overlapping the study as described by [40]. The grid contained 2117,818 cells, each approximately 2.449 × 2.449 km (∼6 km²), covering the locations of 4856 soil samples.

### Environmental data

#### Raster sources and selection

We identified and manually downloaded rasters of publicly available datasets covering the conterminous United States that represented environmental variables with potential influence on soil geochemistry [5,[41], [42], [43]], including soil properties, climate, topography, and land use (Table 1). We considered cation exchange capacity but excluded due to missing values throughout the southeast United States.

**Table 1:** Attribute data of publicly available environmental rasters used in our workflow to model geochemical distributions in the soil of the conterminous United States.

**Variable**	**Units**	**Resolution (m)**	**Source**
**Soil Properties**			
organic matter	kg/m^2^^a^	800	[25]
pH (0–25 cm)	NA^b^	800	[25]
saturated hydraulic conductivity (0–25 cm)	µm/s^c^	800	[25]
percent clay (0–25 cm)	NA^b^	800	[25]
**Climate**			
temperature	C° d	800	[44]
precipitation	mm^e^	800	[44]
**Topography**			[44]
elevation	m f	800	[44]
slope	degrees	800	[44]
**Land Use**			
cultivated crops^g^	NA^b^	30	[47,48]

a kilograms per meter squared.

b not applicable.

c micrometers per second.

d degrees Celsius.

e millimeters.

f meters.

g National Land Cover Database class 82.

#### Soil chemical and physical properties

Rasters of soil chemical and physical properties were derived from the United States Department of Agriculture (USDA)–National Cooperative Soil Survey (SSURGO), supplemented with State Soil Survey data where necessary [25]. Aggregation involved thickness-weighted averages of soil horizons (either 0–25 cm or all horizons), area-weighted averages of components within map units, and area-weighted averages of all map units within each grid cell [25]. Where available, we used rasters of soil chemical and physical properties for soil horizons ranging from 0 to 25 cm to correspond with the depth of the samples collected in the USGS soil survey.

Chemical and physical soil properties we identified as being potentially important predictors of trace elements and minerals included: 1) organic matter (om), the weight of decomposed plant and animal residues <2 mm; 2) pH, a measure of soil acidity or alkalinity; 3) saturated hydraulic conductivity (Ksat), indicating the ease of water movement through saturated soil pores; and 4) percent clay, the proportion of mineral particles <0.002 mm in the <2 mm soil fraction [25].

#### Climatic variables

Temperature and precipitation rasters represented 30-year normals (1991–2020) (Table 1; [44]) and were modeled by parameter elevation regression on independent slopes model (PRISM) using a digital elevation model as the predictor grid [44].

#### Topography

We obtained elevation rasters from PRISM (Table 1 [44]). Slope was derived from the elevation raster using previously established methods [45] and the terra package [46]. Elevation represents mean above-sea-level height, while slope captures mean elevation change per grid cell (Table 1).

#### Land use

We prepared landcover rasters from the Annual National Land Cover Database (NLCD; Table 1 [47,48]) for years corresponding with the years the soil data were collected (2007–2010) to represent the proportion of cultivated crops (NLCD class 82) within each sampling grid cell, using area-weighted means [40]. We selected cultivated crops because fertilizers from anthropogenic sources may influence soil geochemistry [49].

### Process rasters and extract spatial data

#### Project and resample rasters

We projected precipitation, temperature, elevation, and slope rasters to the sampling grid’s coordinate system and resampled each raster to match the sampling grid’s resolution using a template raster we created. We aggregated the binary agriculture raster to calculate the mean proportion of crops per grid cell and then reprojected it to save computation time. We did not reproject or resample the organic matter, ksat, pH, and percent clay rasters because these rasters were already in the projected coordinate system.

#### Crop and align rasters

We cropped all rasters to the extent of the sampling grid and aligned them to have consistent projection, resolution, and spatial extent. After verifying alignment, we saved each raster for use in modeling.

#### Extract environmental data for each soil sample

We used the exactextractr::exact_extract function [50] to calculate the mean value of each environmental variable within each grid cell and linked the results to the corresponding soil samples using sf::st_join (join = st_intersects) function [38,39]. If a soil sample overlapped multiple grid cells and two duplicates were produced, we randomly removed one record for each using base functions in R. We also removed four soil samples that contained one or two NA values for om, ksat, pH, or clay due to missing data in the original rasters.

#### Packages and dependencies

We conducted all processing of the environmental data using terra, sf, and exactextractr packages [38,39,46,50].

## Script 2.0 – Predict mineral soil distributions lognormal

### Overview of mesh construction process

We constructed models for each of the trace elements and fitted the models using R-INLA with the INLA-SPDE method [[10], [11], [12], [13]]. When using INLA-SPDE, a mesh is used to approximate the underlying spatial process as a spatial random field [13]. The mesh is essentially a discretization of the spatial domain, breaking it down into a network of small, interconnected triangles [13]. These triangles define a finite-dimensional approximation of the continuous spatial domain, which is necessary for computational efficiency when working with Gaussian random fields [13].

The mesh components and construction using INLA-SPDE have been previously described [12,31,[52], [53]]. Briefly, the mesh nodes (vertices) represent the locations where the spatial field is evaluated, and the edges (connections between nodes) define the spatial relationships between these points [12]. In R-INLA, the max.edge parameter controls the maximum length of the edges of the inner triangles in the mesh [12]. Smaller max.edge values produce finer meshes whereas larger values produce coarser meshes. The mesh boundary defines the boundary of the study area and spatial extent of the mesh [12]. The offset parameter in the outer region of the mesh specifies the minimum difference between the nodes and the boundary and creates a buffer around the inner mesh to avoid a boundary effect near the edges of the domain [12]. The cutoff parameter helps prevent the creation of numerous small triangles in areas where data points are densely clustered [12].

To generate the mesh, we first defined a spatial domain of the study area, which was the same for all trace elements modeled. Next, using methods described by [52,53], we constructed a coarse mesh for inference and implemented the models. We then constructed a fine mesh that was unique for each trace element to conduct the rest of the analysis, including model validation, selection of priors and generating the predictive risk surface. Using a coarse mesh reduces computational demands and is typically adequate for inference purposes, whereas employing a fine mesh allows for more precise estimation of model parameters and a higher-resolution representation of the spatial process [51,53]. We fitted the models with the coordinates of the data in kilometers (km) instead of meters (m) to avoid large numbers in computations, improve convergence, make parameters more interpretable, and simplify prior specification. The specific steps in the script are further described below.

#### Prepare spatial domain boundary of mesh

For the spatial domain of the mesh, we used the boundary of the conterminous United States (US) and generated it by modifying methods by [40]. Briefly, we loaded data of the US states using a function that downloads the geometries of the states as a simple feature (sf) object using the sf package [38,39]. We then specified specific states and territories to be removed so that the number of states was reduced to 48, removed the internal state boundaries from the US states outline, and removed spatial data that extended beyond the US national shoreline, such as islands in oceans or Great Lakes. We then simplified the boundary of the US to reduce complexity while retaining the overall shape, reprojected the coordinate system, converted the geometry from m to km, and ensured the geometries were valid.

#### Remove locations outside of spatial domain boundary of mesh

**Fig. 1:**
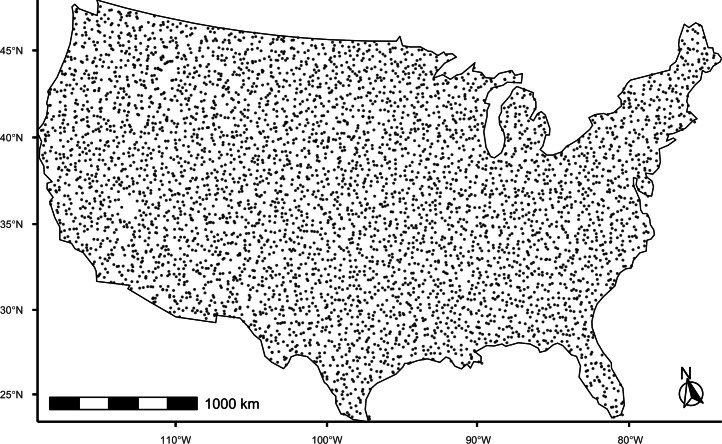
Locations of soil survey locations for trace element modeling of the conterminous US, consisting of a final dataset of 4833 to 4834 locations.

Before performing the calculations, we loaded the soil survey dataset. For each trace element modeled, we removed the outlying observation previously specified with “NA”. We then converted the coordinates from m to km and removed 18 soil survey locations that fell outside of the US boundary using the sf: st_within function [38,39]. The modeled dataset for each trace element consisted of a total of either 4833 or 4834 soil survey locations (Fig. 1).

#### Construct coarse mesh for model selection

We constructed the coarse mesh as described by [51]. We used the spatial extent of the soil survey data to determine the max.edge and offset parameters of the triangles for the inner and outer domains of the mesh. For the offset parameter of the outer mesh, we calculated the total spatial extent in the x-direction between the maximum and minimum values of the soil survey locations and divided by three. For the value of the max.edge parameter of the inner triangles of the mesh, we calculated the total spatial extent as described above and divided by 12. This produced a mesh with a resolution that was approximately one-twelfth of the spatial extent of the study area, which was suggested to be a good starting point by [54] for modeling annual precipitation in the conterminous United States and also found to be suitable by [51] for modeling chronic wasting disease in deer using a Bernoulli distribution at a relatively smaller spatial extent. For the max.edge parameter for the outer mesh, we multiplied the edge length by five to save computing time. For the cutoff value, we divided the value of the max.edge parameter used for the inner triangles by five.

**Fig. 2:**
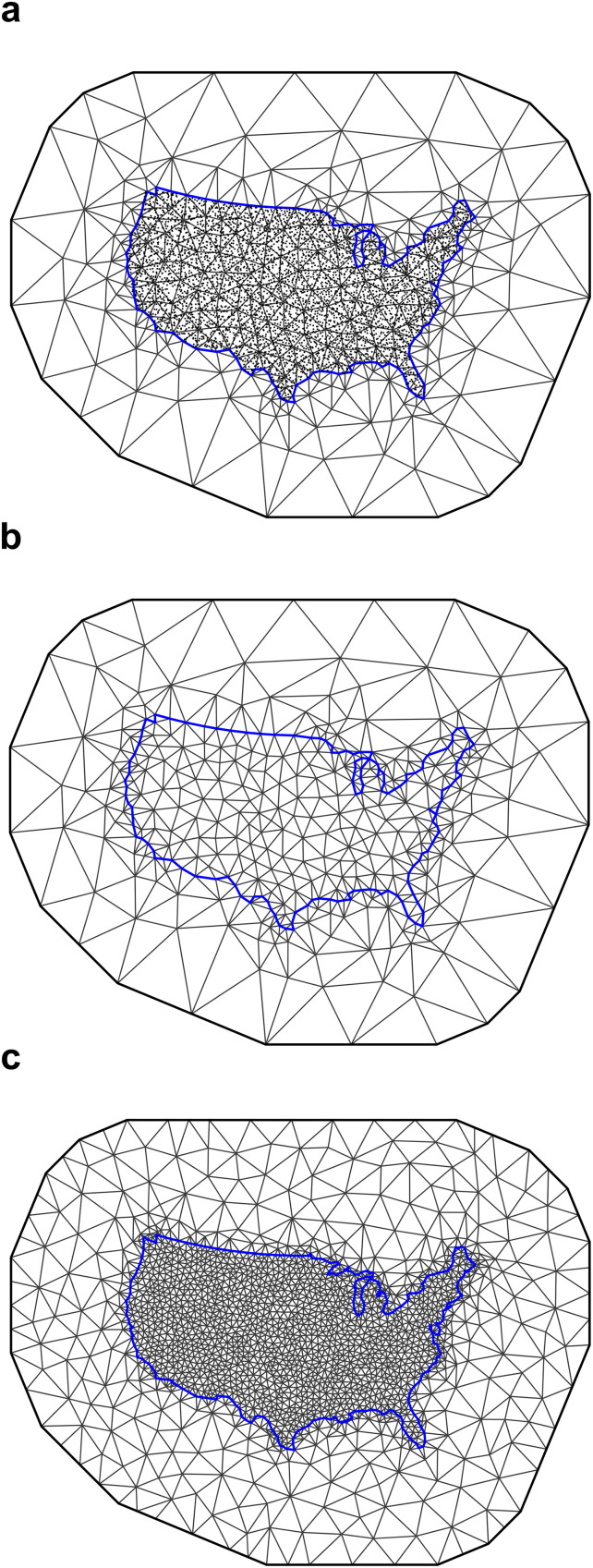
Spatial meshes used in R-INLA models of trace element distributions. (a) Coarse mesh with soil survey points overlaid. (b) Coarse mesh without soil survey points overlaid. (c) Fine mesh without points overlaid for the top model of each element (cobalt shown), constructed by setting maximum triangle edge lengths to one-tenth of the mean spatial range from the corresponding model fitted using the coarse mesh.

We then plotted the coarse mesh with and without points to visualize (Figs. 2a–b). Coarse mesh evaluation was performed by examining the triangles for consistency in size and shape, aiming for triangles that were as regular and uniform as possible [31].

#### Explore response distributions and prepare data for spatial modeling

We investigated correlations among the covariates and found precipitation to be highly correlated (Pearson’s |r|>0.7 [55]) with pH; thus, we excluded precipitation in the models. We then plotted histograms of the data and determined the data were skewed for each of the trace elements. Because the values for each trace element were all positive, we considered “gamma” and “lognormal” distributions [56]. We scaled the covariates for modeling to ensure that covariates contributed comparably to the model and to help with convergence and stability in R-INLA. We then built an R-INLA data stack to fit the models, which included the response, covariates, and spatial field.

#### Select priors

The SPDE model represents a Gaussian random field using a Matérn covariance function parameterized by: the range (p), which controls the spatial correlation distance; and marginal standard deviation (σ), which controls the variability of the spatial field [31].

The priors for p and σ are based on penalized complexity (PC) priors [57,58], which reduce overfitting and improve predictive performance by penalizing deviations from the base model [33]. In R-INLA, PC priors for p and σ are defined as:

(1) Prob(p<u)=α

(2) $$\begin{array}{c} \mathrm{Prob}\;\left(\sigma \;>\;u\right)\;=\;\alpha \end{array}$$

where α is a probability, 0<α<1, and u is the user-defined threshold value of the parameter (p and σ, respectively in expressions 1 and 2 above) [31].

To determine u in the prior for p*,* we calculated the total spatial extent in the x-direction between the maximum and minimum values in km as described above and divided by two, which we defined as *range_ob* (Table 2). We selected priors for p and σ that allowed for spatial correlation and variability to be guided by the data (Table 2).

**Table 2:** Penalized complexity prior specifications for the spatial field. The range prioris defined using Prob(parameter < threshold = α, and the marginal standard deviation (Sigma) prior is defined as Prob(parameter > threshold) = α, where α is a probability 0<α<1. Descriptions explain the expected effect of the prior.

**Range prior** (p)	**Sigma prior** (σ)	**Interpretation**
P(p<range_model)=0.5	P(σ>0.5)=0.5	Moderately informative prior used as the baseline for models fitted using fine mesh; allows the data to guide spatial correlation while discouraging extreme values and maintaining flexibility in the spatial field. range_model was calculated using the range of top model fitted using coarse mesh.
P(p<500)=0.5	P(σ>0.1)=0.5	Prior favors short-range spatial correlation and low variability, encouraging localized spatial structure.
P(p<range_ob)=0.5	P(σ>1)=0.5	Weakly informative prior that allows the data to determine spatial correlation and variability,permitting higher variance. range_ob was calculated from data points.
P(p<range_model)=0.2	P(σ>0.5)=0.99	Prior favors long-range spatial correlation while strongly restricting variability, emphasizing broad-scale smoothness with limited local variability. range_model was calculated using the range of top model fitted using coarse mesh.

We used baseline weakly informative priors for the fixed effects and intercept (Table 3), which are less informative than the default priors in R-INLA. By default, R-INLA uses a normal prior for the fixed effects, with mean 0 and precision 0.001, and a prior for the intercept with mean and precision 0 [33]. To evaluate the sensitivity of posterior estimates to prior specification, we varied the precision of the priors of the fixed effects across a range of values representing both more and less informative assumptions (Table 3).

**Table 3:** Prior specifications (mean, precision) used to assess sensitivity of posterior estimates to precision of the priors for both intercept and fixed effects within the same model. Precision is defined as the inverse of variance, with lower precision corresponding to weaker prior influence.

**Intercept prior**	**Fixed effect prior**
Normal (0, 0.0001)^a^	Normal (0, 0.0001)^a^
Normal (0, 0.01)	Normal (0, 0.01)
Normal (0, 0.000001)	Normal (0, 0.000001)
Normal (0, 0.01)	Normal (0, 0.000001)
Normal (0, 0.000001)	Normal (0, 0.01)

a Weakly informative baseline prior used in primary analysis.

#### Fit baseline and automated models, and select top predictive model

We fit baseline models for both gamma and lognormal distributions to evaluate inclusion of the spatial effect and a state boundary random effect. The state boundary random effect was included to account for local variation and was modeled with an independent and identically distributed (iid) effect using the default gamma prior on precision (shape = 1, rate = 0.00005) [33]. To compare base models, we calculated Watanabe-Akaike information criterion (WAIC), deviance information criterion (DIC), and mean logarithmic conditional predictive ordinate (LCPO), with lower values indicating better model fit [59,60]. LCPO values were derived from conditional predictive ordinates as described by [61]. The spatial effect and state boundary random effect had better model fit based on these criteria, so we included these variables in each model with the covariates.

For each trace element, we constructed models *a priori* with combinations of soil and chemical physical properties, topography, land use, and climatic variables for a total of 29 models for each of the modeled distributions. We listed the models in a csv file, imported into R, and automatically fit them using R-INLA. In selecting the best performing models, we prioritized WAIC over DIC for model selection because WAIC provides a fully Bayesian measure of predictive accuracy by averaging over the entire posterior distribution, whereas DIC evaluates model fit using the deviance at a single point estimate of the parameters (posterior mean) and may underestimate uncertainty in hierarchical or latent-variable models [62].

#### Select top model from each distribution, refit with fine mesh for validation, and compare metrics

For the top model of each mineral, following [53] we constructed a fine mesh (Fig. 2c) by adjusting the max*.edge* parameter of the inner triangles to one-tenth of the mean range of the spatial field, which was obtained from the corresponding top model results using the coarse mesh. We used the mean range of the spatial field of the top model as the baseline prior for the range of the spatial effect for models fitted using the fine mesh (Table 2).

To evaluate whether a gamma or lognormal distribution better described the data, we compared the top model from each distribution using DIC, WAIC (Table S1), and LCPO, alongside two goodness-of-fit evaluations, which included Bayesian posterior predictive checks and leave-one-out (LOOCV) and leave-group-out (LGOCV) cross-validation implemented in R-INLA [64]. Goodness-of-fit evaluations were conducted following previously described methods [51,63,64].

**Fig. 3:**
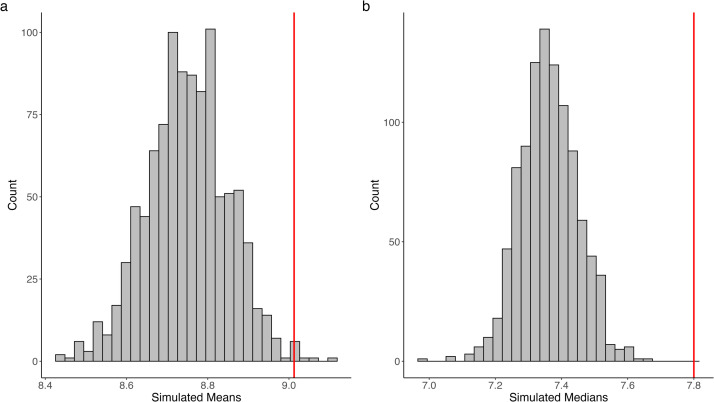
(a) Histogram of simulated means and (b) histogram of simulated medians from 1000 posterior samples of the lognormal model for cobalt. Observed values are indicated by red lines; values falling within the simulated distributions as in (a) indicate that the model adequately captures the corresponding summary statistic.

For posterior predictive checks, we simulated 1000 samples from the posterior distribution of the best-fitting gamma and lognormal models (based on WAIC) fit using the fine mesh. We summarized these simulations by computing the mean, median, variance, and 90th quantile for each sample, and calculating the lower and upper bounds of the 95 % credible interval (CI) for each summary statistic (Table S2). Observed values were then compared to these intervals to assess coverage. To aid interpretation, side-by-side histogram plots of simulated means and medians were produced, with observed values indicated by a red line. If the observed value fell within the distribution of simulated values, the model was considered to adequately capture that summary statistic (Figs. 3a–b).

We evaluated predictive performance as described previously by [51]. Briefly, we used LOOCV and LGOCV, where either a single observation or small groups of six, eight, or ten observations were temporarily removed [64]. Predictive performance was summarized using log-predictive scores, which quantify the probability of the observed data under the model's predictive distribution, with higher scores indicating better fit. We compared expected log predictive densities across cross-validation levels by plotting the results with a 1:1 reference line using the abline function (intercept = 0, slope = 1) [27], representing perfect agreement between levels.Consistent scores and results across different LOOCV and LGOCV levels indicated stable model performance that was not overly sensitive to the removal of individual or small groups of observations.

We compared top models using both gamma and lognormal distributions. Summaries of DIC, WAIC, LCPO, and goodness-of-fit assessments, including LOOCV and LGOCV, generally indicated better predictive performance for the lognormal distribution. In posterior predictive checks of the mean, median, variance, and lower and upper bounds of the 95 % CI , the gamma distribution occasionally performed better; however, no distribution achieved a perfect fit. Considering all metrics together, we selected the lognormal distribution for the remainder of the analysis.

#### Perform sensitivity analyses of mesh, priors, and covariates

**Fig. 4:**
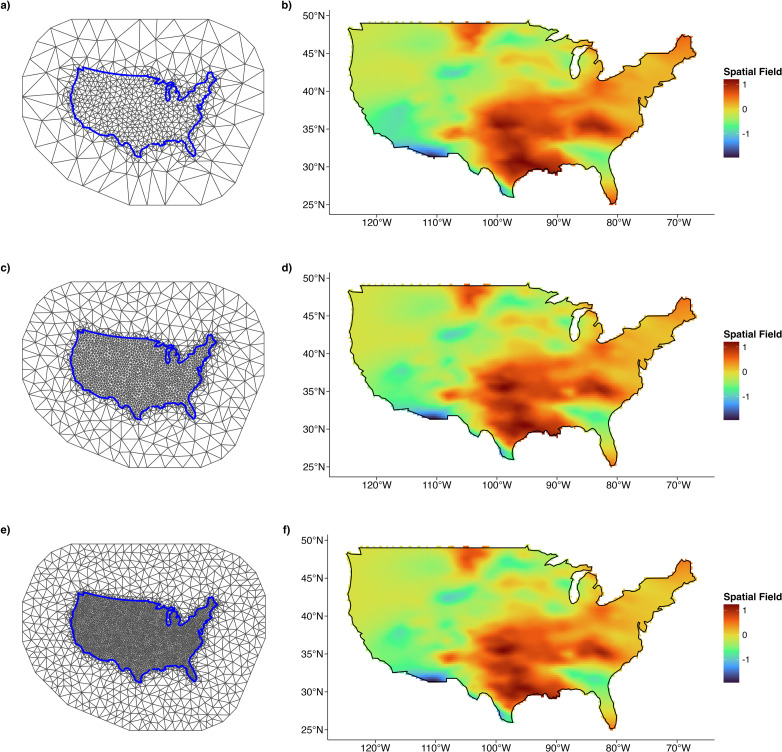
a) One-fifth, (b) one-tenth (fine mesh), and (c) one-fifteenth meshes used to assess the effect of mesh resolution on spatial model outputs for cobalt. Maximum triangle edge lengths were scaled relative to the estimated spatial range of the top model fitted using the coarse mesh (Fig. 2b), with all other parameters held constant.

To evaluate the sensitivity of spatial model outputs to mesh resolution, we performed a mesh sensitivity analysis for each of the mineral models by comparing the fine mesh, constructed using a max.edge value equal to 1/10 of the estimated spatial range (Fig. 4c), with two alternative meshes that had max-edge values of 1/5 (one-fifth mesh; Fig. 4a) and 1/15 (one-fifteenth mesh; Fig. 4e) of the estimated spatial range. All other mesh construction parameters were held constant to isolate the effect of mesh resolution.

We also assessed the sensitivity of model results to the cutoff parameter used during mesh construction. This parameter influences the minimum allowable distance between observation points in the mesh, and when using a polygon boundary, it also affects how precisely the boundary is captured by the mesh. Using the top-performing model for each mineral, we tested the impact of adjusting the cutoff to 0.5, while holding other mesh parameters constant.

We assessed the sensitivity of priors of the spatial random field, intercept, and fixed effectsby varying their values over several orders of magnitude and comparing to our selected baseline priors (Table 2, Table 3). Across all models, fixed effect estimates remained consistent regardless of mesh resolution, indicating robust inference for covariate effects. However, the one-fifth mesh typically resulted in higher estimates of the spatial range and standard deviation of the spatial field, along with poorer model fit as indicated by elevated WAIC values. In contrast, the fine mesh and one-fifteenth mesh produced nearly identical WAIC scores (e.g., differences <10 units), similar spatial hyperparameter estimates, and comparable spatial field patterns (Figs. 4d and 4f) when plotted.

These results suggest that model inference was sensitive to overly coarse meshes but stabilized once the mesh resolution sufficiently captured local spatial structure. Accordingly, we adopted the fine mesh for all final models, balancing spatial resolution with computational efficiency. Mesh sensitivity analyses by [51] also indicated that using a mesh finer than 1/10 the estimated spatial range offered little additional improvement.

Model performance metrics (DIC and WAIC), as well as the posterior means of the fixed effects and spatial hyperparameters, were compared across the meshes with varying cutoff parameters. In general, the results remained stable, indicating that model inference was not sensitive to the choice of cutoff within the tested range. This suggests that the selected cutoff value adequately balanced computational efficiency with spatial precision in representing the study boundary.

When evaluating sensitivity of priors, we compared resulting DIC, WAIC, posterior mean estimates, and the mean range and standard deviation of the spatial field. Because these metrics changed only minimally across prior settings, we considered the model results to be robust to the choice of priors.

If notable differences had been observed, we included code to further validate the model using alternative meshes and priors, allowing the sensitivity analysis to guide model refinement or further exploration.

#### Visualize and interpret spatial field

In the INLA-SPDE framework, the spatial random field w(s) is a latent Gaussian process indexed by spatial location s, approximated by a Gaussian Markov random field on the mesh [11,13,65]. This field captures residual spatial dependence in the outcome not explained by covariates [66]. Under lognormal or gamma likelihoods, the spatial field enters the linear predictor on the log scale of the response. Consequently, its effect is multiplicative on the original response scale: a positive value of w(s) increases the expected outcome by a factor of exp⁡{w(s)}, while a negative value decreases it by that factor [30]. A value near zero indicates little residual spatial influence [30]. The spatial field’s correlation structure is described by two hyperparameters: the range, which indicates the distance over which residual spatial correlation effectively diminishes (conventionally where correlation drops to ∼ 0.1), and the marginal variance (or standard deviation), which quantifies the magnitude of residual spatial variation [65].

Using the model results obtained from the fine mesh for the top model of each trace element, we extracted the spatial field estimates from the model, defined the grid resolution based on the limits of the mesh, and then projected the posterior mean of the spatial random field onto a grid for visualization (Fig. 4d).

#### Predict to grid using fine mesh

To generate predictions at unsampled locations, we imported and standardized each raster by its mean and standard deviation, consistent with the covariate processing. We masked the rasters to match the distribution of missing values, converted them to data frames, and transformed each into an sf object [38,39] using the projected coordinate system. These sf objects were combined using Reduce function [27] based on spatial proximity. Coordinates were then extracted, converted from m to km to align with the covariate scale, and the final data frame was converted back to an sf object in the projected coordinate system.

Using R-INLA, we constructed a stack of the covariates as previously described and created a corresponding prediction stack using the sf object derived from the rasters. We then joined the data and prediction stacks and fit the model with control.compute = list(config = TRUE) [31] to obtain the full set of model outputs used to generate the predictions at new locations.

#### Generate predictive rasters of posterior mean and uncertainty

We extracted the posterior mean predictions and lower and upper bounds of the 95 % CI from the model output and created a template raster using the extent and projection of the study area boundary, with a resolution of approximately 2.4 km. Rasterization was performed with background = NA using the terra package [46] to preserve missing values. We then masked the raster to the study area boundary to exclude values outside the region of interest and converted the raster coordinates from kilometers back to meters for final raster and visualization.

#### Plot geochemical distributions for visualization on a continuous scale and with ten percentiles

**Fig. 5:**
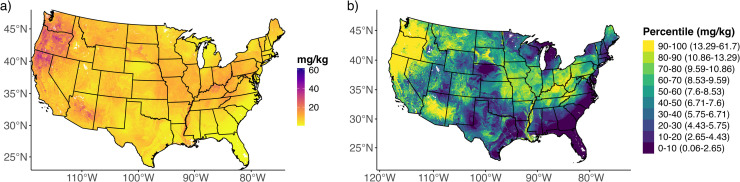
(a) Posterior mean of cobalt concentrations using a lognormal distribution on a continuous scale. (b) Posterior means of cobalt grouped into ten percentiles (10–100 %) with labels showing the corresponding raw value ranges, facilitating interpretation of predicted absolute and relative concentrations. The corresponding lower and upper bounds of the 95 % credible interval are shown in Fig. S1c-d.

For visualization of the posterior distributions, we mapped the posterior mean of each element on a continuous scale, which is the scale of the rasters (cobalt; Fig. 5a and Fig. S1a; other elements Figs. S2a – S6a). We also plotted the lower and upper bounds of the 95 % CI of each trace element on a continuous scale (not shown).

To facilitate interpretation across large spatial extents, we also generated maps that binned the posterior mean values into ten percentiles (10–100 %), which was the approach used for USGS interpolated maps [5]. Each percentile was also labeled with the corresponding raw value range, allowing both relative and absolute concentrations to be interpreted (cobalt; Fig. 5b and S1b; other elements Figs. S2b – S6b).

We also produced percentile maps of the lower and upper bounds of the 95% CI using the same percentile breaks as the posterior mean to maintain consistency across visualizations and allow simultaneous visualization of spatial patterns in trace element estimates and the uncertainty in these predictions across the study region (Figs. S1c–d – S6c–d).

Percentile maps of the posterior mean and lower and upper bounds of the 95% CI were plotted using a color-blind–accessible palette to more clearly distinguish between categories (Figs. S1a–d – S6a–d). In addition, the posterior mean itself was displayed separately using a color palette similar to the USGS geochemical distribution maps (Figs S7a–S12a), enabling direct visual comparison with existing interpolated maps by [5]. For each trace element, we also plotted the posterior mean of the gamma distribution (Figs S7b–S12b) for comparison with the lognormal distribution (Figs S7a–S12a).

#### Packages

In this script, we used base functions and the following packages: dplyr (data manipulation [36]), sf, terra, stars, rmapshaper, tigris, and fmesher (processing spatial data [36,38,39,46,[67], [68], [69]]); R-INLA (mesh generation, model fitting, and predictions [[10], [11], [12], [13]], and ggplot2 and gridExtra (visualization; [70,71]). R-INLA is not distributed through CRAN and must be installed from the project’s repository.

## Method validation

We validated the final models for each trace element using Bayesian posterior predictive checks and LOOCV and LGOCV, as described above. While neither the gamma nor lognormal distribution provided a perfect fit according to the posterior predictive checks, the gamma distribution occasionally better captured specific aspects of the observed data (e.g., mean, median, or variance). However, because our primary focus was predictive performance, we selected the lognormal distribution, which generally showed superior predictive ability based on DIC, WAIC, LCPO, and cross-validation results.

To assess the spatial plausibility of predictions, we compared the percentile maps of the posterior mean for each trace element from the lognormal and gamma models (Figs S7a–S12a and Figs S7b–S12b) with the corresponding USGS interpolated maps. Although exact agreement was not expected due to differences in modelling approaches and data sources, we visually evaluated whether the predicted surfaces broadly captured similar spatial patterns, including regional hotspots, gradients, and general magnitude ranges. The surfaces appeared consistent as the predictions fell within the same order of magnitude and reflected comparable spatial trends to the USGS maps.

## Limitations

Although INLA-SPDE provides an efficient and flexible framework for spatial modeling, several limitations are noted. Model performance can be influenced by the density and spatial distribution of sampling locations [72]. In our study, covariate data were complete, but the relatively low density of sampling sites may reduce the model’s ability to capture fine-scale spatial variation, even though evenly spaced sites help mitigate broad-scale bias. The spatial random field assumes stationarity, which may not hold for heterogeneous geology, potentially leading to over- or under-smoothing [73].

As with any model, simplifications are inherent, and no model perfectly captures complex geochemical patterns. While we recognize that soil element concentrations are strongly influenced by geologic parent material [3], a detailed national-scale geologic map of the United States [74] is highly heterogeneous and consists of categorical units that would require substantial subjective aggregation and simplification to incorporate into the predictive models. Instead, we used soil physicochemical, topographical, climatic, and landscape variables as predictors because these factors are shaped by weathering and pedogenesis, processes that integrate the effects of parent material, climate, and soil development [75,76]. These predictors have been shown to capture variation in soil nutrient concentrations [77], thereby partially integrating parent material effects through pedogenic processes.

In our analysis, the lognormal distribution was generally selected for its superior predictive ability, but the gamma distribution occasionally fit observed data better in posterior predictive checks, suggesting it may capture local variation more accurately. Thus, predicted rasters based on the lognormal model may under-or over-estimate concentrations relative to gamma-based predictions. Users can interpret results in the context of these considerations and, where appropriate, supplement outputs with domain knowledge or additional data sources, such as local soil surveys, geological maps, or independent trace element measurements, to evaluate whether predicted patterns are reasonable for a particular region.

Posterior predictive checks (Table S2) indicate that these models do not capture all features of the element distributions perfectly, which is expected given the complexity of soil geochemistry; nonetheless, the inference is likely robust enough to reveal broader spatial and compositional patterns. While we focused on lognormal and gamma distributions, more advanced distributions that may provide better model fit are also available but may require customization and careful validation. More advanced users may modify this code to explore alternative or more flexible distributional assumptions to better capture specific features of the data where needed. For localized studies, users may consider using gamma, or another suitable distribution, if posterior predictive checks or domain expertise indicate it better reflects local mineral patterns. Additional covariates, finer-resolution sampling, or regional studies could further improve predictive performance.

Despite these limitations, the resulting rasters, to our knowledge, represent the most comprehensive and high-resolution distributions of trace element distributions of the conterminous United States available to date, providing a valuable resource. These outputs can serve as a baseline for future studies, allow comparison with other spatial data products, and support informed decision-making in environmental, ecological, and agricultural applications.

## Ethics statements

This method did not involve studies with living things.

## CRediT author statement

**Kristin Bondo:** Conceptualization, Methodology, Software, Data Curation, Formal Analysis, Validation, Visualization, Writing – Original Draft, Writing – Reviewing & Editing. **Tiffany Wolf:** Resources, Supervision, Project administration, Funding acquisition, Writing – Reviewing and Editing. **W. David Walter:** Conceptualization, Methodology, Data Curation, Resources, Software, Visualization, Writing – Reviewing and Editing.

## Related research article

None.

## Declaration of competing interest

The authors declare that they have no known competing financial interests or personal relationships that could have appeared to influence the work reported in this paper.

## Data Availability

The code used in the workflow is available from [26], and downloadable rasters of the posterior mean of each trace element modeled with a lognormal distribution are available from [29].

## Appendix Supplementary materials

Image, application 1
