# Supplementary material for: Generating geochemical and mineralogy distributions of soil in the conterminous United States using Bayesian hierarchical spatial models
Source: MethodsX. 2026 Feb 19;16:103836. doi: 10.1016/j.mex.2026.103836 (PMC12961220; doi:10.1016/j.mex.2026.103836)
Supplement: Supplementary file 1 [file mmc1.docx]

**Generating geochemical distributions of soil in the conterminous United States using R-INLA.**

Kristin J. Bondo^1,2^, Tiffany M. Wolf^1,2^, and W. David Walter^3*^

^1^ Department of Veterinary Population Medicine, University of Minnesota, St. Paul, Minnesota 55108, USA

^2^ Minnesota Center for Prion Research and Outreach (MNPRO), University of Minnesota, St. Paul, Minnesota 55108, USA

^3^ U.S. Geological Survey, Pennsylvania Cooperative Fish and Wildlife Research Unit, The Pennsylvania State University, University Park, PA, 16802, USA

**Supplemental**

Any use of trade, firm, or product names is for descriptive purposes only and does not imply endorsement by the U.S. Government.

Table S1: Models and variables used to generate the predictive distribution of trace elements in the soil of the conterminous United States. For each element, the distribution family (Gamma or Lognormal), the set of covariates included in the model, and the Widely Applicable Information

Criterion (WAIC) are reported

| **Mineral** | **Distribution** | **Model^1^** | **WAIC^2^** |
| --- | --- | --- | --- |
| Cobalt | gamma | clay + om + ksat + ph + elev + slope | 26149.60 |
| Cobalt | lognormal | clay + om + ksat + ph + elev + slope | 25899.58 |
| Copper | gamma | clay + ph + elev + slope + tmean + agric | 33329.21 |
| Copper | lognormal | clay + om + ksat + ph + elev + slope + tmean + agric | 32665.14 |
| Iron | gamma | clay + om + ksat + ph + elev + slope | 12291.81 |
| Iron | lognormal | clay + ph + elev + slope + tmean + agric | 11831.20 |
| Manganese | gamma | clay + om + ksat + ph + elev + slope + agric | 68033.57 |
| Manganese | lognormal | clay + om + ksat + ph + elev + slope | 67753.86 |
| Selenium | gamma | tmean | -4673.354 |
| Selenium | lognormal | clay + om + ksat + ph + elev + slope + agric | -6484.993 |
| Zinc | gamma | clay + om + elev + slope + tmean + agric | 46520.55 |
| Zinc | lognormal | clay + om + ksat + ph + elev + slope + agric | 44497.96 |

^1^ clay – percent clay; om – organic matter; ksat – saturated hydraulic conductivity; ph – pH; elev – elevation.

^2^ Lower values of WAIC indicate better predictive performance among models of each trace mineral.

Table S2: Posterior predictive check results for the predicted distribution of trace elements of the conterminous United States using models from Table S1. For each element, observed values of the mean, median, variance, and 90th percentile are compared to the lower and upper bounds of the 95% credible interval (CI) of posterior predictive simulations. “True” indicates the observed value falls within the 95% CI; “False” indicates it falls outside.

| **Element** | **Distribution** | **Statistic** | **Observed** | **Simulated Lower CI** | **Simulated Upper CI** | **Covered** |
| --- | --- | --- | --- | --- | --- | --- |
| Cobalt | gamma | mean | 9.01 | 8.82 | 9.21 | True |
|  |  | median | 7.80 | 6.96 | 7.34 | False |
|  |  | variance | 55.76 | 51.69 | 51.69 | False |
|  |  | 90^th^ quantile | 16.44 | 16.44 | 16.44 | False |
|  |  |  |  |  |  |  |
| Cobalt | lognormal | mean | 9.01 | 8.57 | 8.94 | False |
|  |  | median | 7.80 | 7.21 | 7.52 | False |
|  |  | variance | 55.76 | 42.31 | 56.56 | True |
|  |  | 90^th^ quantile | 56.56 | 15.44 | 16.66 | True |
|  |  |  |  |  |  |  |
| Copper | gamma | mean | 18.29 | 17.59 | 18.31 | True |
|  |  | median | 14.95 | 13.75 | 14.42 | False |
|  |  | variance | 284.19 | 197.43 | 253.16 | False |
|  |  | 90^th^ quantile | 32.04 | 33.12 | 35.35 | False |
|  |  |  |  |  |  |  |
| Copper | lognormal | mean | 18.29 | 16.98483 | 17.71069 | False |
|  |  | median | 14.95 | 14.08 | 14.71 | False |
|  |  | variance | 284.19 | 145.63 | 195.55 | False |
|  |  | 90^th^ quantile | 32.04 | 30.14 | 32.31 | True |
|  |  |  |  |  |  |  |
| Iron | gamma | mean | 2.17 | 2.13 | 2.21 | True |
|  |  | median | 1.97 | 1.79 | 1.87 | False |
|  |  | variance | 1.99 | 2.17 | 2.63 | False |
|  |  | 90^th^ quantile | 3.78 | 3.93 | 4.16 | False |
|  |  |  |  |  |  |  |
| Iron | lognormal | mean | 2.17 | 2.10 | 2.17 | True |
|  |  | median | 1.97 | 1.84 | 1.92 | False |
|  |  | variance | 1.99 | 1.82 | 2.31 | True |
|  |  | 90^th^ quantile | 3.78 | 3.67 | 3.91 | True |
|  |  |  |  |  |  |  |
| Manganese | gamma | mean | 617.66 | 599.06 | 629.48 | True |
|  |  | median | 499.00 | 461.52 | 490.31 | False |
|  |  | variance | 276283.19 | 238705.14 | 288181.24 | True |
|  |  | 90^th^ quantile | 1185.00 | 1218.32 | 1307.30 | False |
|  |  |  |  |  |  |  |
| Manganese | lognormal | mean | 617.66 | 574.85 | 603.12 | False |
|  |  | median | 499.00 | 472.24 | 497.33 | False |
|  |  | variance | 276283.19 | 188554.47 | 248984.69 | False |
|  |  | 90^th^ quantile | 1185.00 | 1058.71 | 1137.41 | False |
|  |  |  |  |  |  |  |
| Selenium | gamma | mean | 0.311 | 0.293 | 0.306 | False |
|  |  | median | 0.200 | 0.209 | 0.220 | False |
|  |  | variance | 0.0961 | 0.0653 | 0.080 | False |
|  |  | 90^th^ quantile | 0.650 | 0.605 | 0.646 | False |
|  |  |  |  |  |  |  |
| Selenium | lognormal | mean | 0.311 | 0.280 | 0.293 | False |
|  |  | median | 0.200 | 0.210 | 0.21 | False |
|  |  | variance | 0.0962 | 0.0464 | 0.0614 | False |
|  |  | 90^th^ quantile | 0.650 | 0.536 | 0.578 | False |
|  |  |  |  |  |  |  |
| Zinc | gamma | mean | 63.73 | 62.13 | 64.61 | True |
|  |  | median | 58.50 | 52.67 | 55.27 | False |
|  |  | variance | 2557.42 | 1686.96 | 1990.92 | False |
|  |  | 90^th^ quantile | 105.50 | 114.79 | 121.98 | False |
|  |  |  |  |  |  |  |
| Zinc | lognormal | mean | 63.73 | 60.33 | 62.49 | False |
|  |  | median | 58.50 | 55.00 | 57.14 | False |
|  |  | variance | 2557.42 | 1229.08 | 1514.19 | False |
|  |  | 90^th^ quantile | 105.50 | 101.38 | 107.56 | True |


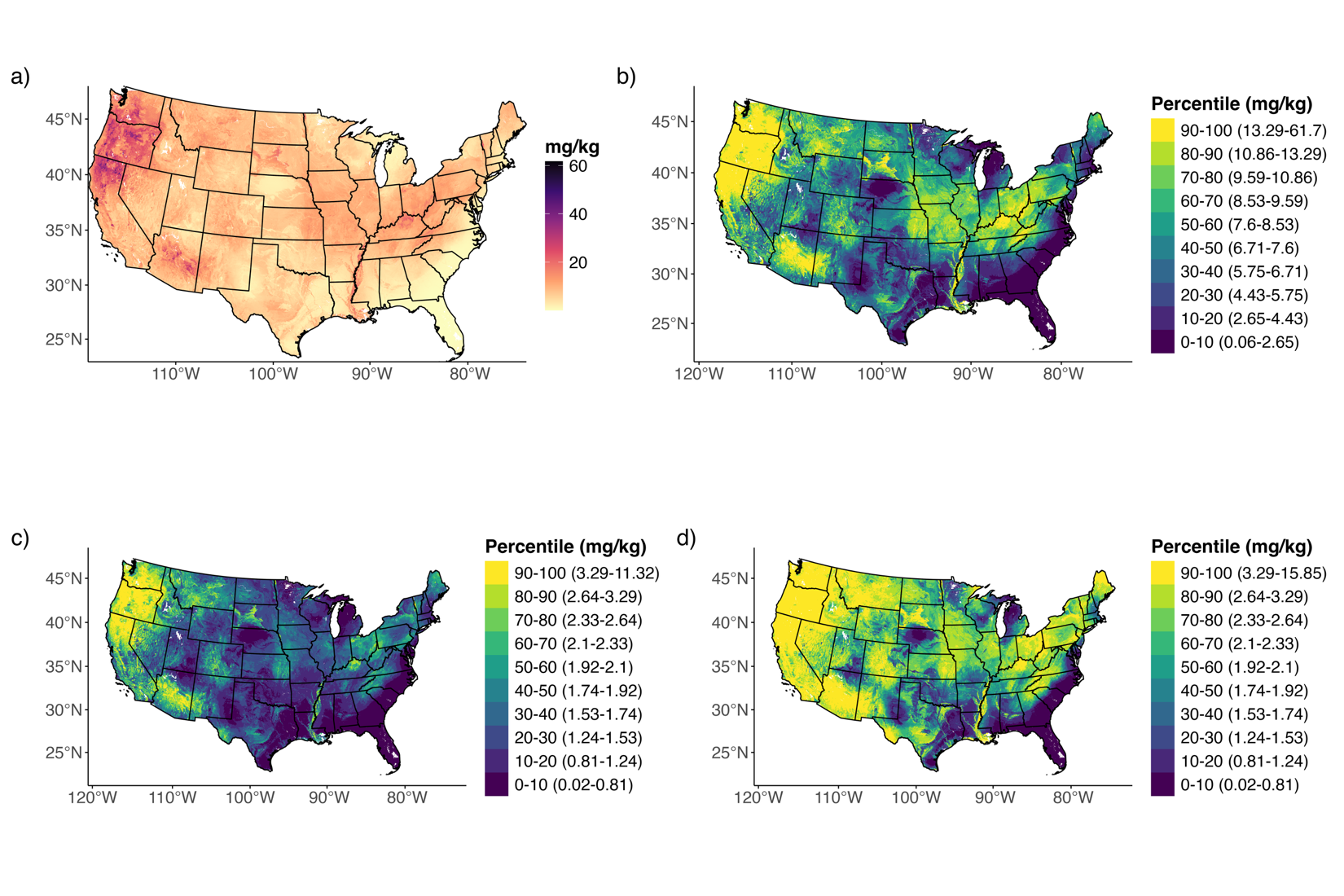


**Figure S1.** (a) Continuous predictive surface of cobalt concentrations (mg/kg) of the conterminous United States. (b) Posterior mean (c) lower 95% credible interval bound (0.025 quantile), and (d) upper 95% credible interval bound (0.975 quantile) are shown using percentiles (0–10, 10–20, …, 90–100) of the posterior mean to highlight spatial patterns.


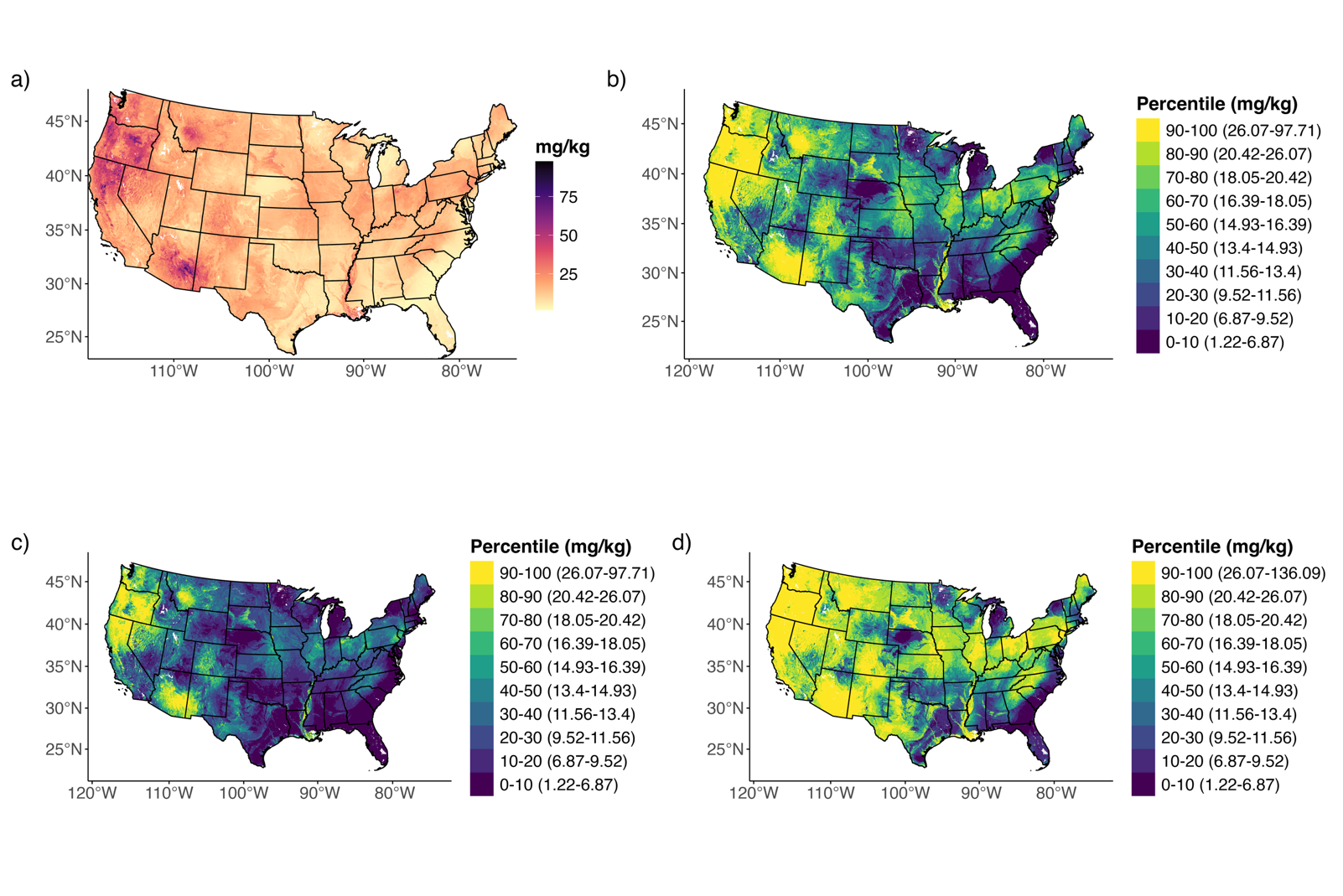


**Figure S2.** (a) Continuous predictive surface of copper concentrations (mg/kg) of the conterminous United States. (b) Posterior mean (c) lower 95% credible interval bound (0.025 quantile), and (d) upper 95% credible interval bound (0.975 quantile) are shown using percentiles (0–10, 10–20, …, 90–100) of the posterior mean to highlight spatial patterns.


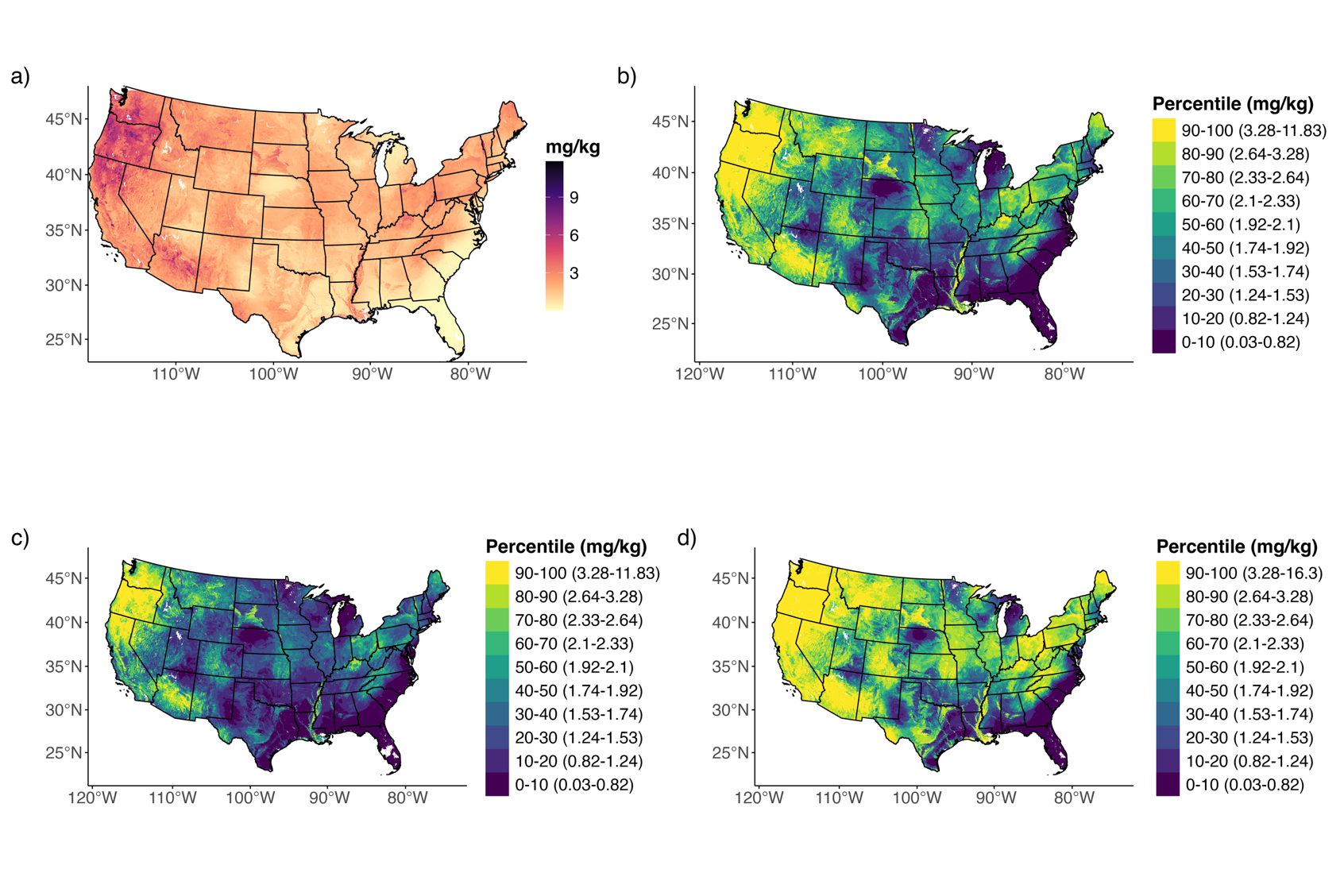


**Figure S3.** (a) Continuous predictive surface of iron concentrations (mg/kg) of the conterminous United States. (b) Posterior mean (c) lower 95% credible interval bound (0.025 quantile), and (d) upper 95% credible interval bound (0.975 quantile) are shown using percentiles (0–10, 10–20, …, 90–100) of the posterior mean to highlight spatial patterns.


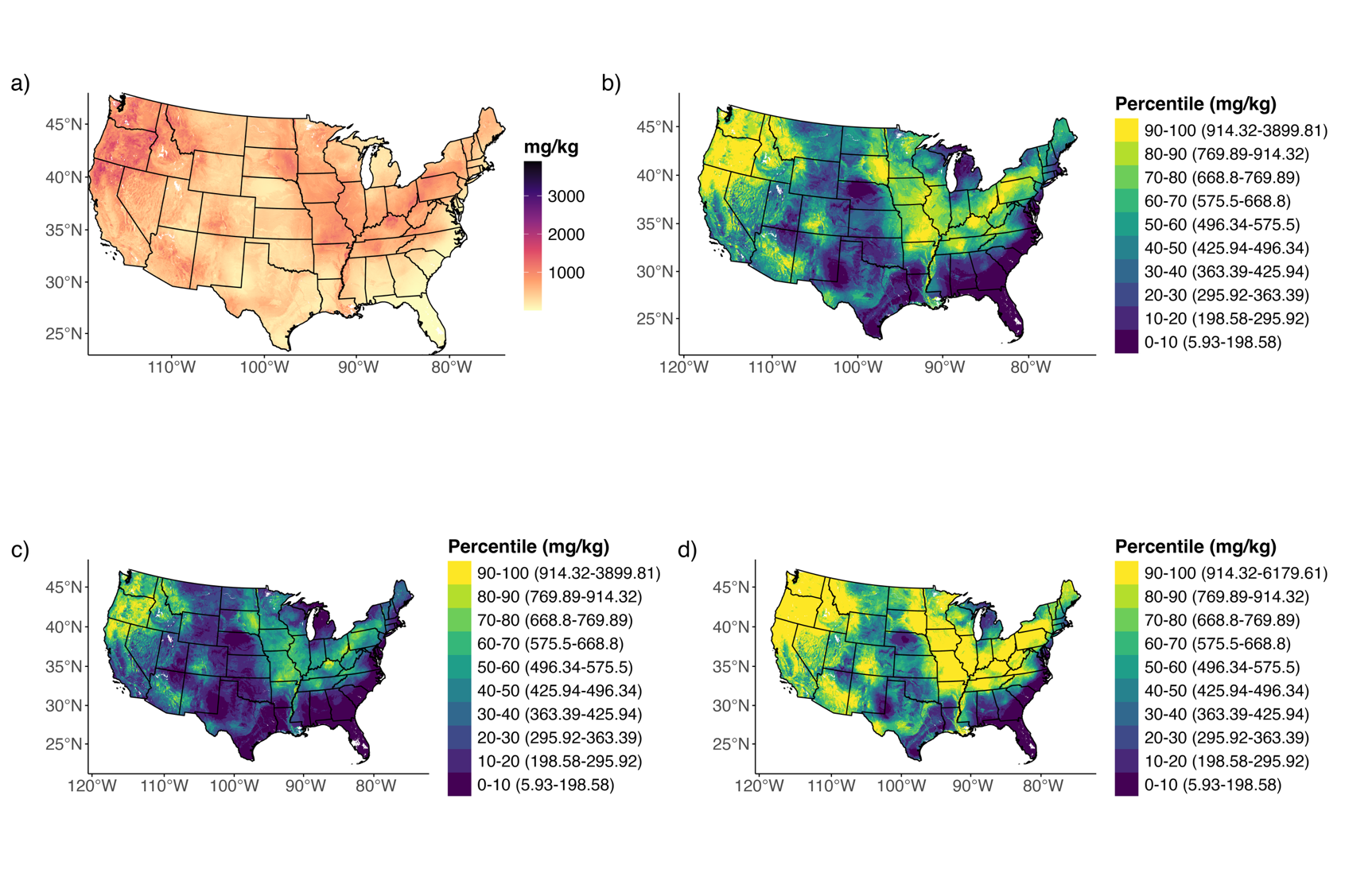


**Figure S4.** (a) Continuous predictive surface of manganese concentrations (mg/kg) of the conterminous United States. (b) Posterior mean (c) lower 95% credible interval bound (0.025 quantile), and (d) upper 95% credible interval bound (0.975 quantile) are shown using percentiles (0–10, 10–20, …, 90–100) of the posterior mean to highlight spatial patterns.


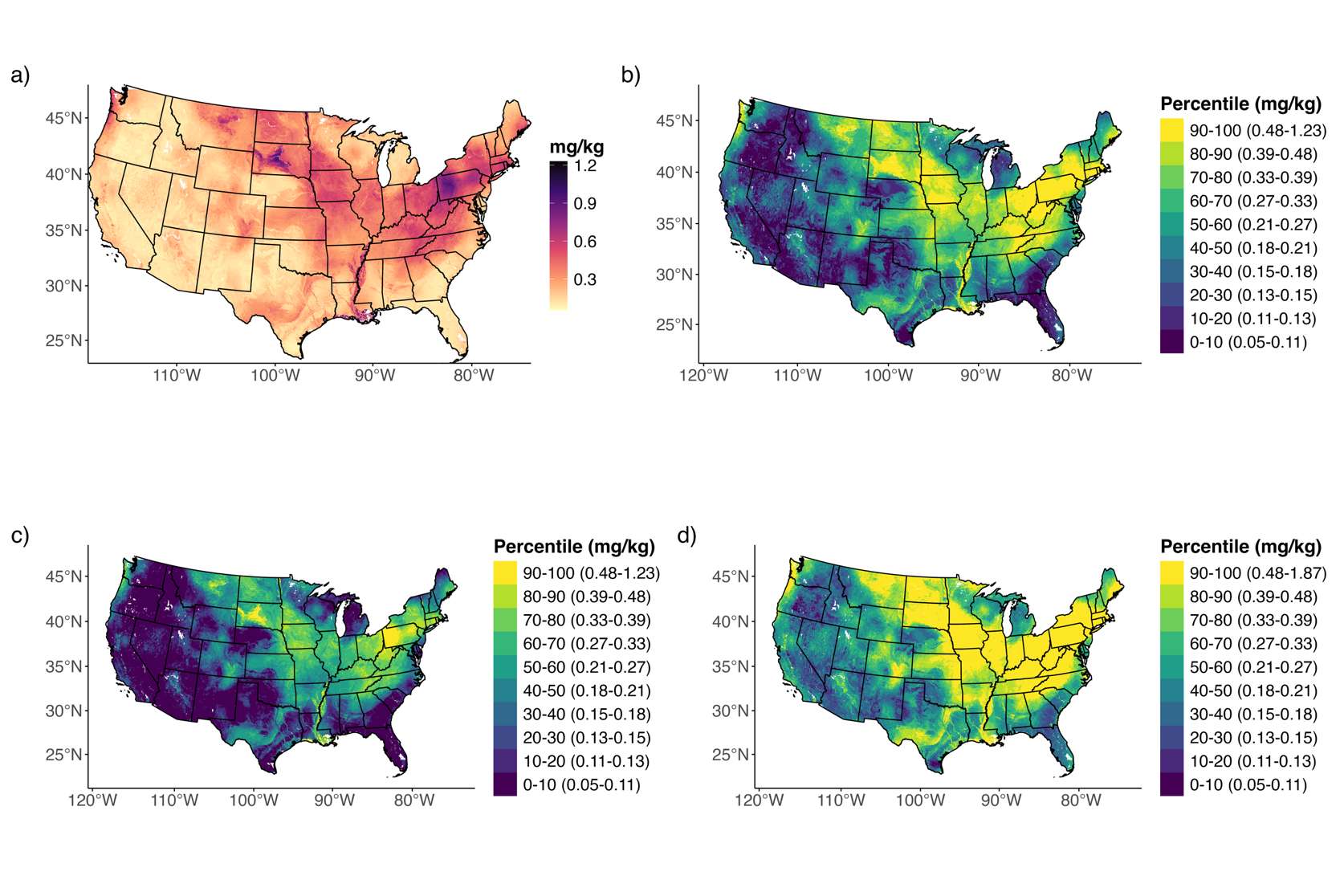


**Figure S5.** (a) Continuous predictive surface of selenium concentrations (mg/kg) of the conterminous United States. (b) Posterior mean (c) lower 95% credible interval bound (0.025 quantile), and (d) upper 95% credible interval bound (0.975 quantile) are shown using percentiles (0–10, 10–20, …, 90–100) of the posterior mean to highlight spatial patterns.


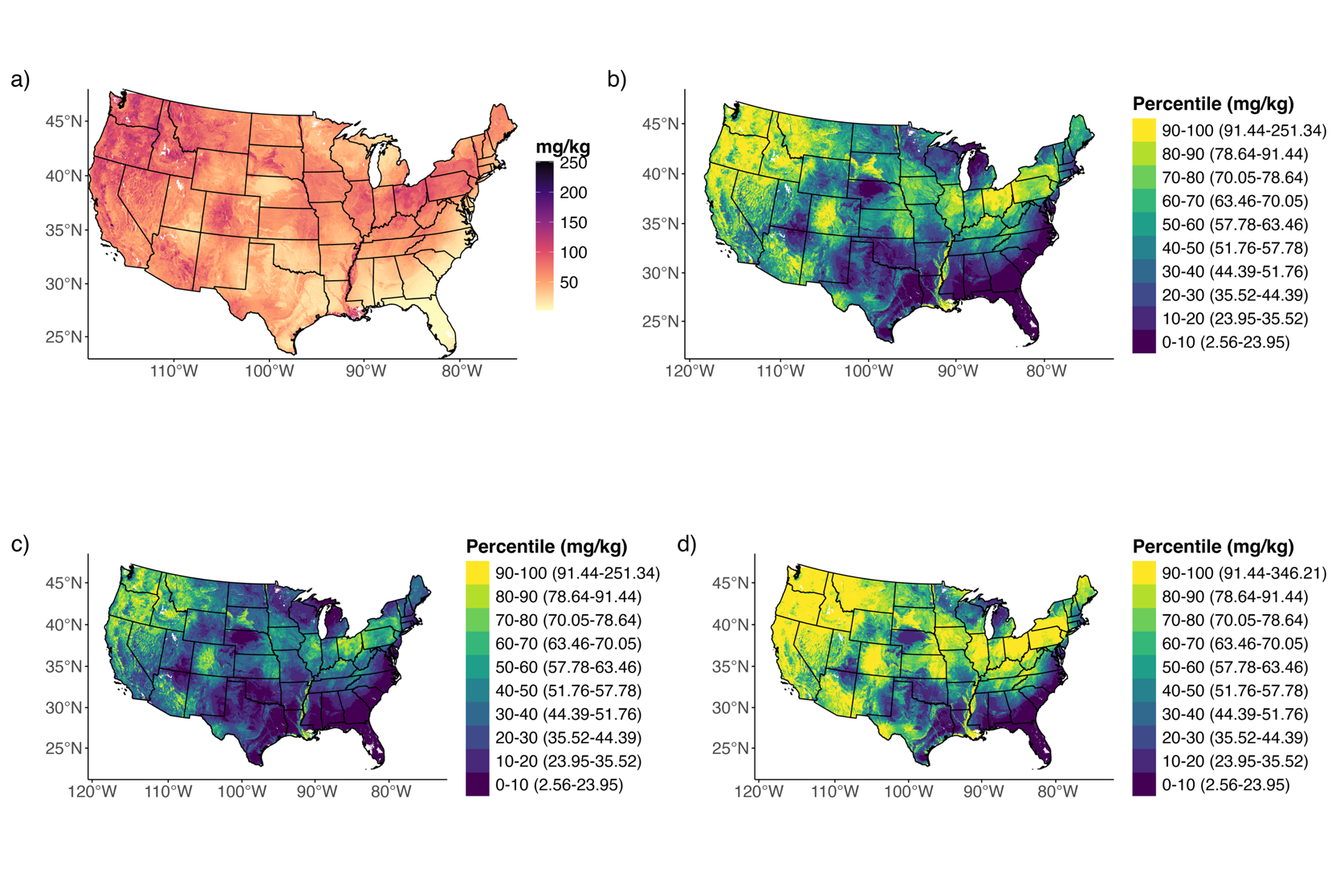


**Figure S6.** (a) Posterior mean of zinc concentrations (mg/kg) of the conterminous United States on continuous scale. (b) Posterior mean (c) lower 95% credible interval bound (0.025 quantile), and (d) upper 95% credible interval bound (0.975 quantile) are shown using percentiles (0–10, 10–20, …, 90–100) of the posterior mean to highlight spatial patterns.


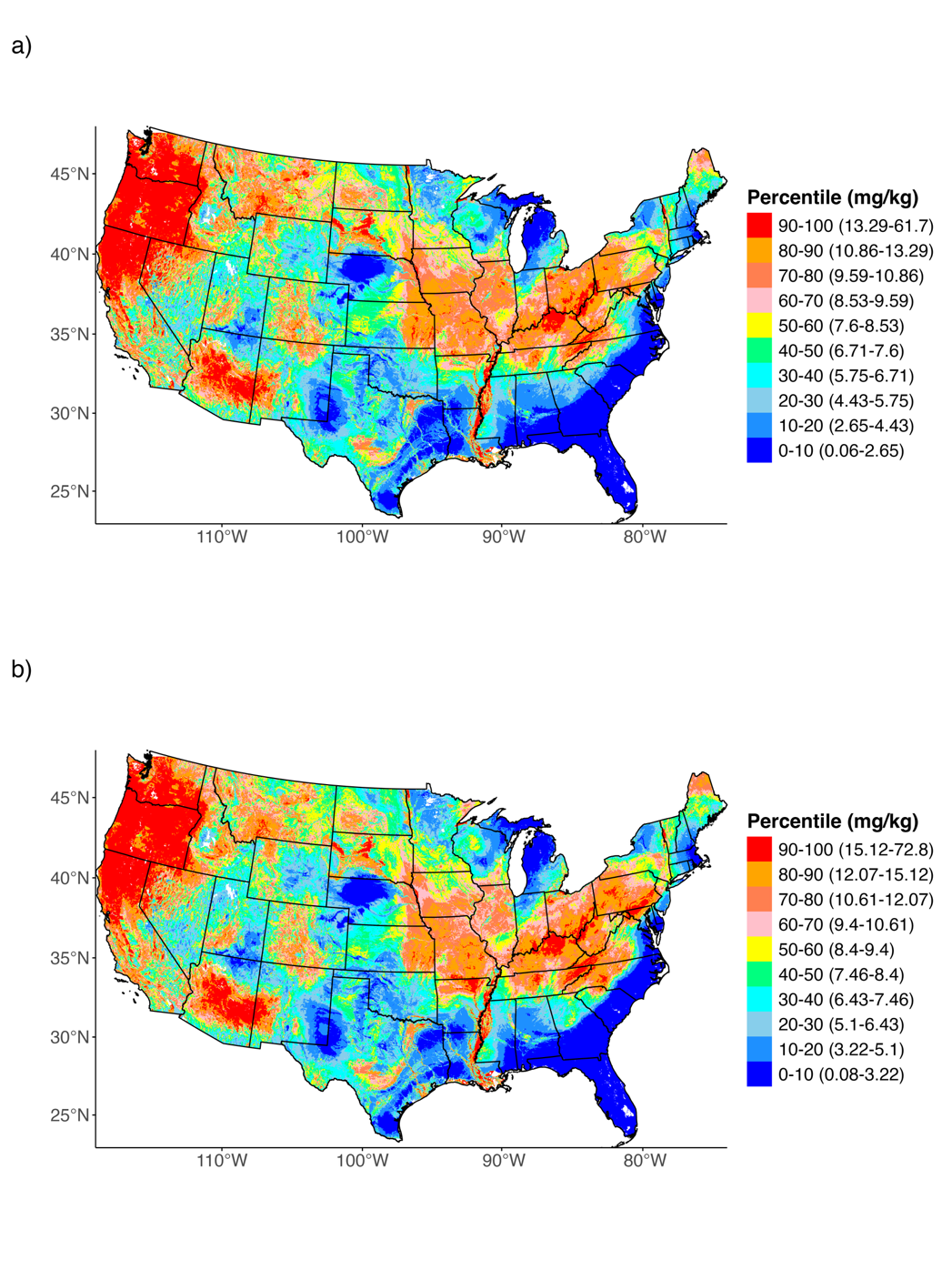


**Figure S7.** Spatial distribution of cobalt concentrations across the conterminous United States. Lognormal model (a) and gamma model (b) posterior means are shown using percentile-based maps to highlight relative spatial patterns. Color scales are consistent across panels to facilitate comparison, and palettes were chosen for accessibility and consistency with geochemical distribution maps from the U.S. Geological Survey.


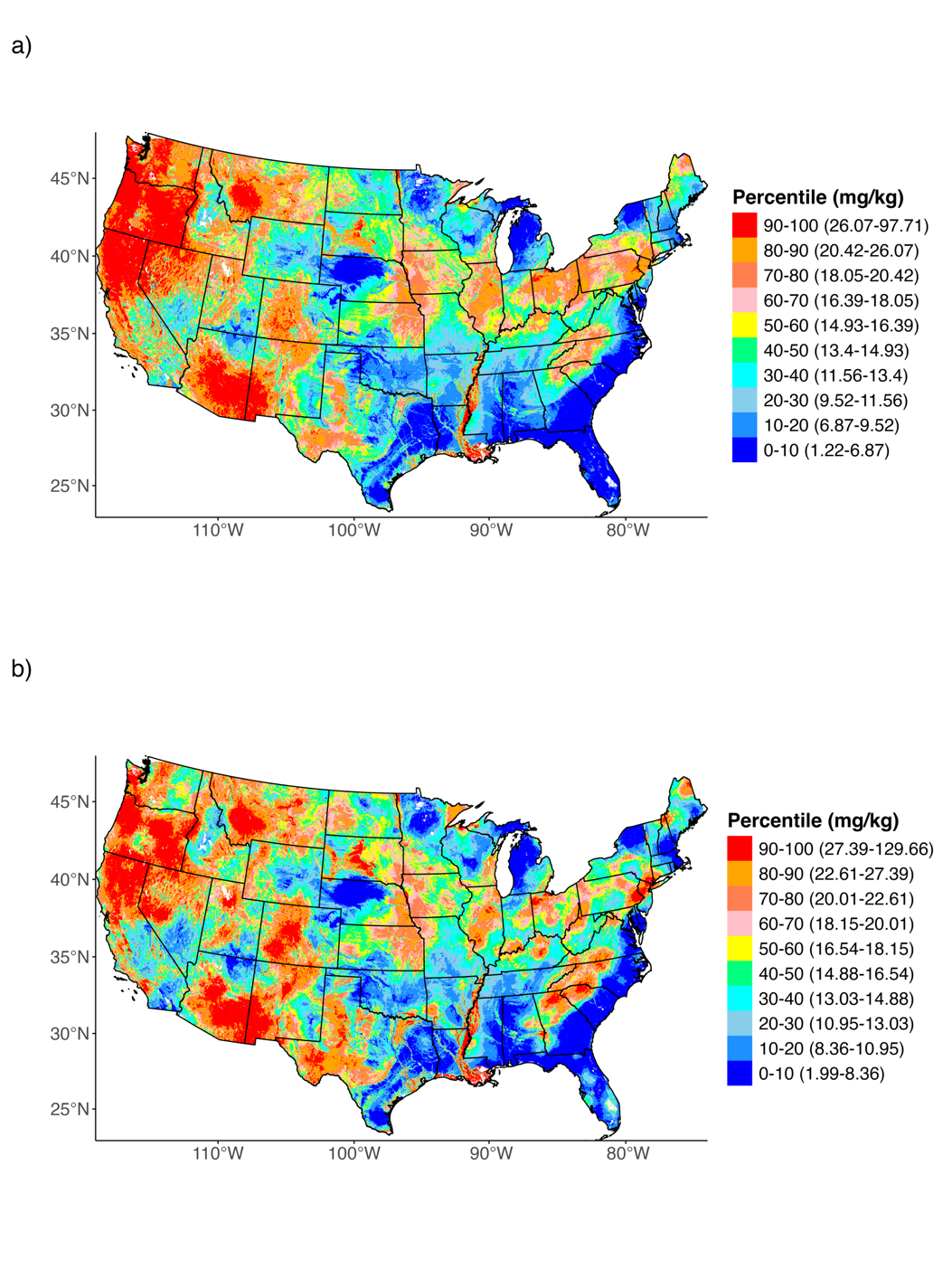


**Figure S8.** Spatial distribution of copper concentrations across the conterminous United States. Lognormal model (a) and gamma model (b) posterior means are shown using percentile-based maps to highlight relative spatial patterns. Color scales are consistent across panels to facilitate comparison, and palettes were chosen for accessibility and consistency with geochemical distribution maps from the U.S. Geological Survey.


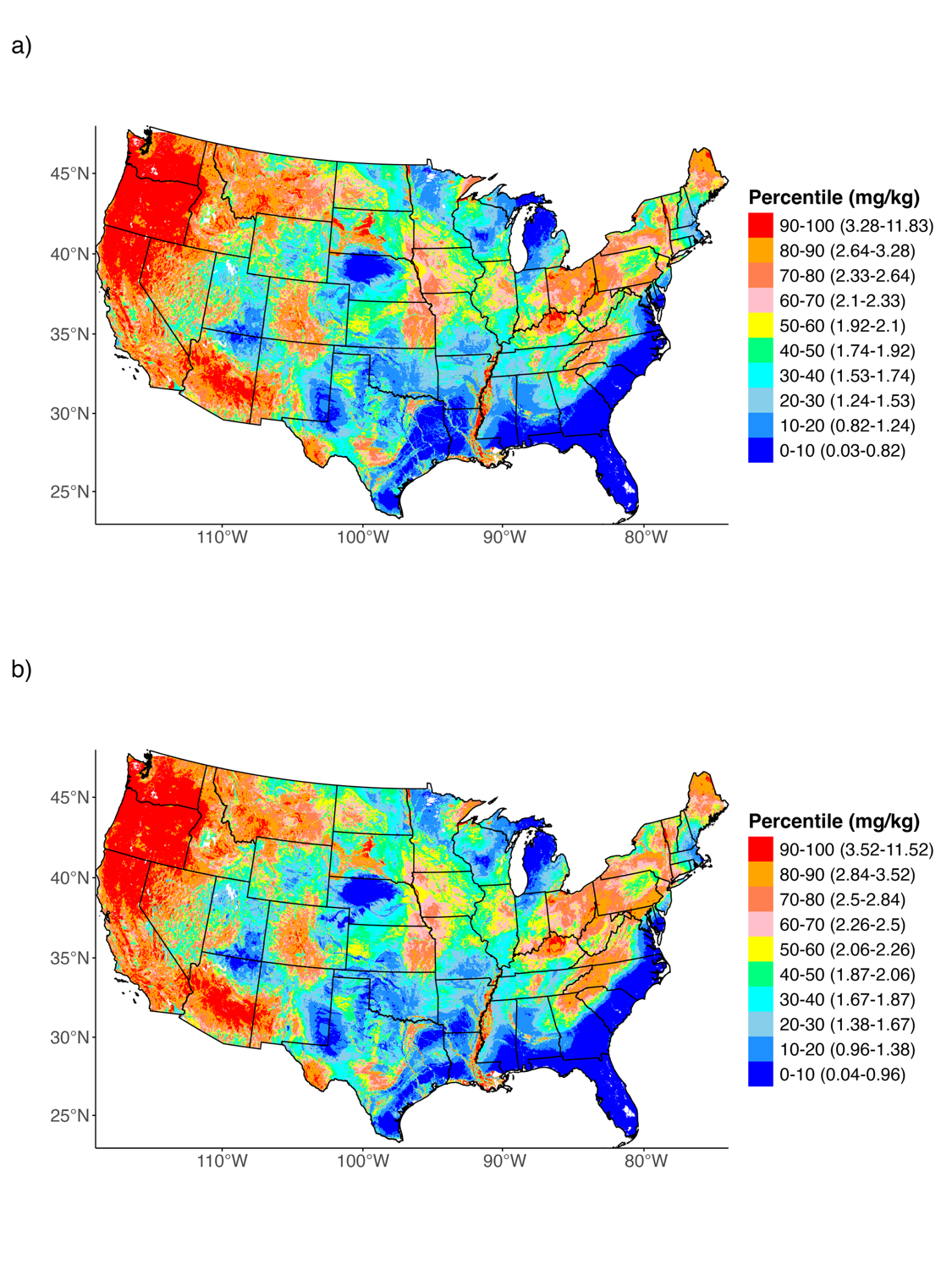


**Figure S9.** Spatial distribution of iron concentrations across the conterminous United States. Lognormal model (a) and gamma model (b) posterior means are shown using percentile-based maps to highlight relative spatial patterns. Color scales are consistent across panels to facilitate comparison, and palettes were chosen for accessibility and consistency with geochemical distribution maps from the U.S. Geological Survey.


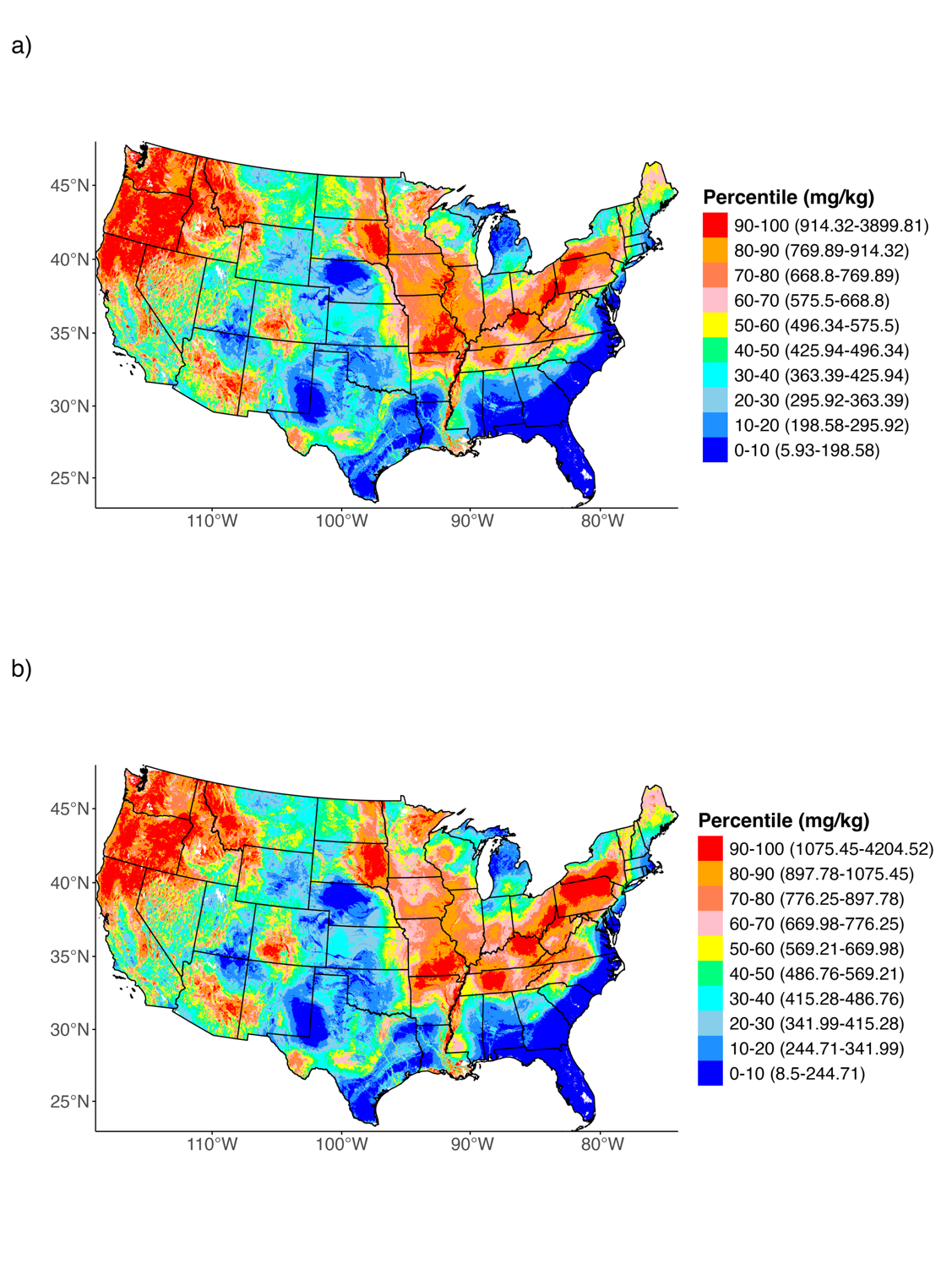


**Figure S10.** Spatial distribution of manganese concentrations across the conterminous United States. Lognormal model (a) and gamma model (b) posterior means are shown using percentile-based maps to highlight relative spatial patterns. Color scales are consistent across panels to facilitate comparison, and palettes were chosen for accessibility and consistency with geochemical distribution maps from the U.S. Geological Survey.


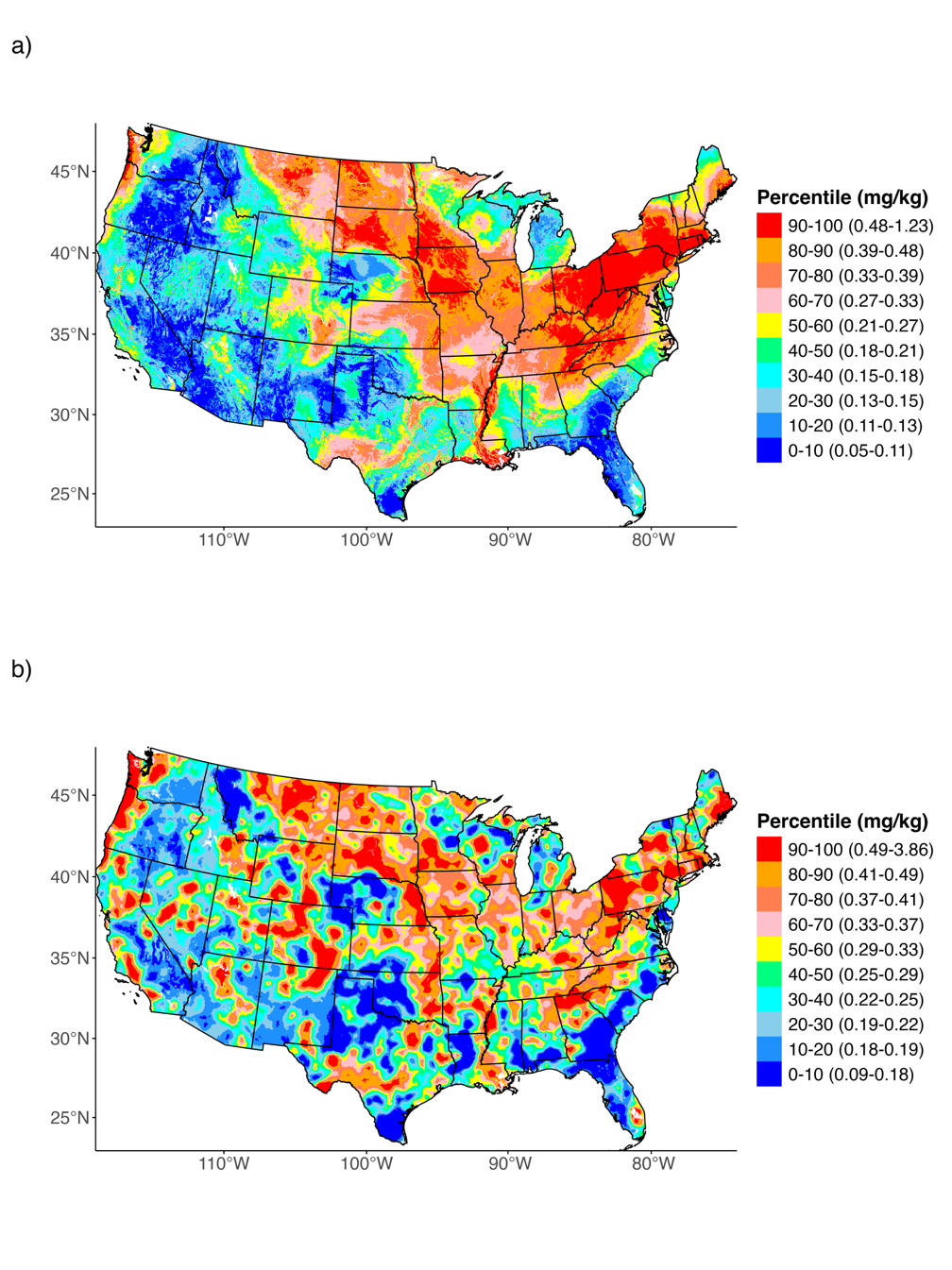


**Figure S10.** Spatial distribution of selenium concentrations across the conterminous United States. Lognormal model (a) and gamma model (b) posterior means are shown using percentile-based maps to highlight relative spatial patterns. Color scales are consistent across panels to facilitate comparison, and palettes were chosen for accessibility and consistency with geochemical distribution maps from the U.S. Geological Survey.


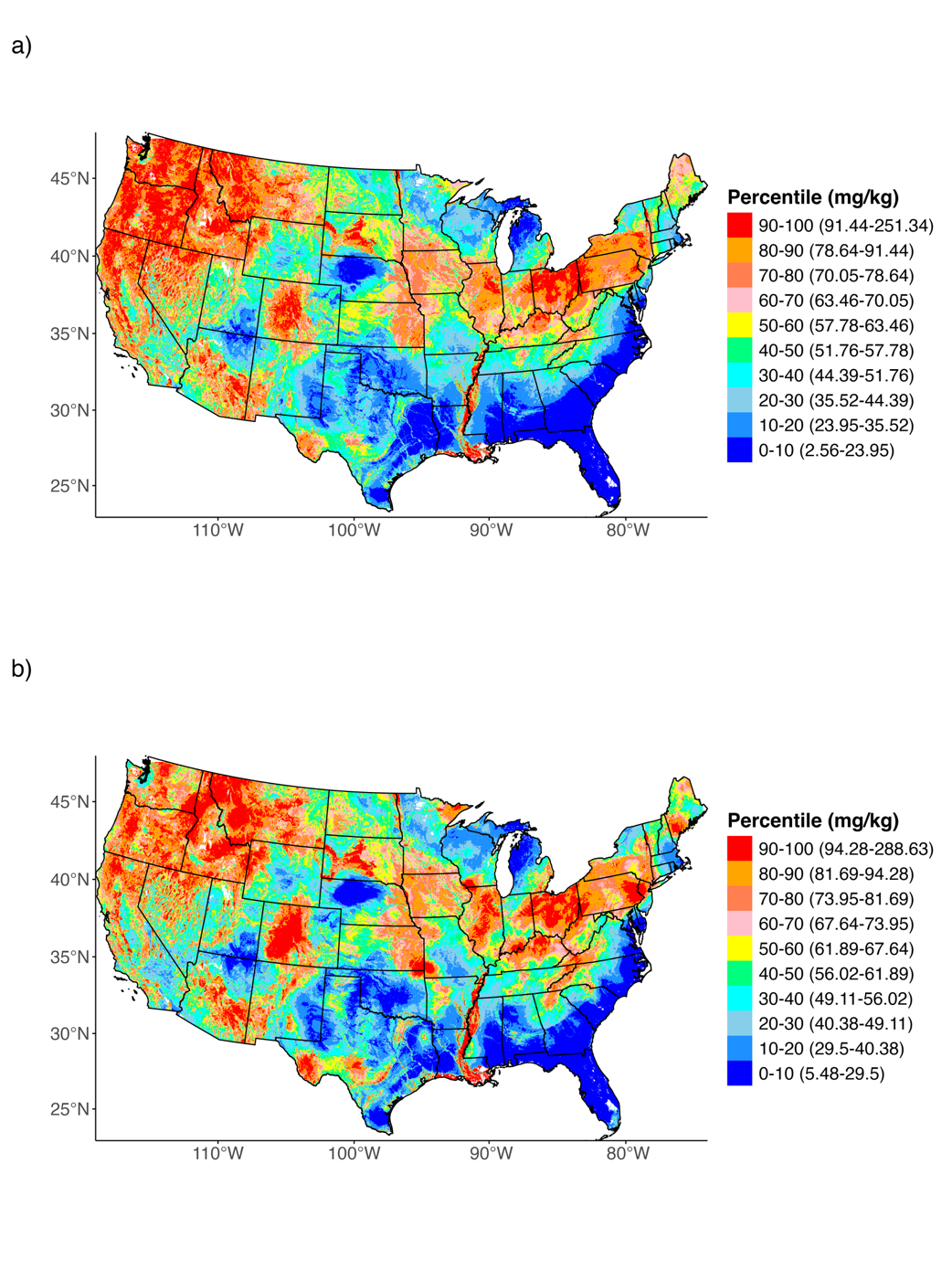


**Figure S10.** Spatial distribution of zinc concentrations across the conterminous United States. Lognormal model (a) and gamma model (b) posterior means are shown using percentile-based maps to highlight relative spatial patterns. Color scales are consistent across panels to facilitate comparison, and palettes were chosen for accessibility and consistency with geochemical distribution maps from the U.S. Geological Survey.
